# Infarto Isolado do Ventrículo Direito – O Ventrículo Direito ainda é o Ventrículo Esquecido?

**DOI:** 10.36660/abc.20200164

**Published:** 2021-02-02

**Authors:** Ana Marques, Inês Cruz, Alexandra Briosa, Isabel João, Sofia Almeida, Hélder Pereira

**Affiliations:** 1 Hospital Garcia de Orta Departamento de Cardiologia Almada Portugal Departamento de Cardiologia, Hospital Garcia de Orta, Almada - Portugal

**Keywords:** Infarto Miocárdio, Doença da Artéria Coronária, Revascularização Miocárdica, Ecocardiografia/métodos, Ressonância Magnética/métodos, Intervenção Coronária Percutânea, Diagnóstico por Imagem

## Introdução

O infarto do miocárdio isolado do ventrículo direito é extremamente raro e é frequentemente silencioso, com apenas 25% dos pacientes desenvolvendo manifestações hemodinâmicas clinicamente evidentes na apresentação.^1^ O manejo atual do infarto agudo do miocárdio baseia-se no diagnóstico imediato e na revascularização imediata.^2^ Aproximadamente 90% dos pacientes que apresentam infarto do miocárdio com supradesnivelamento do segmento ST apresentam estenose ou oclusão da artéria coronária explicativa.^3^ O infarto do miocárdio com artérias coronárias não obstrutivas (MINOCA) deve levar o médico assistente a investigar as causas subjacentes, uma vez que a falha em identificar a causa básica pode resultar em terapia inadequada a esses pacientes.

Descrevemos um caso de infarto do miocárdio isolado do ventrículo direito com exame físico normal, ecocardiogramas transtorácicos normais e doença arterial coronariana não obstrutiva na angiografia coronária, cujo diagnóstico definitivo foi estabelecido por ressonância magnética cardíaca.

## Relato de caso

Um homem branco de 64 anos foi admitido no hospital com histórico de 1 hora de dor torácica anterior opressiva aguda de início súbito, sem outros sintomas associados. Após terapia com nitrato sublingual, o paciente apresentou alívio total da dor. Seu histórico médico incluía hipertensão arterial, dislipidemia e ex-tabagismo.

Na admissão, o paciente estava consciente e hemodinamicamente estável (pressão arterial: 130/70 mmHg e frequência cardíaca: 70 bpm), com apirexia, eupneia e saturação periférica de oxigênio de 99%. Nenhuma alteração na ausculta cardíaca e pulmonar foi observada, e não havia pressão venosa jugular elevada ou edema de membros inferiores. A inspeção abdominal também foi normal.

**Figura 1:**
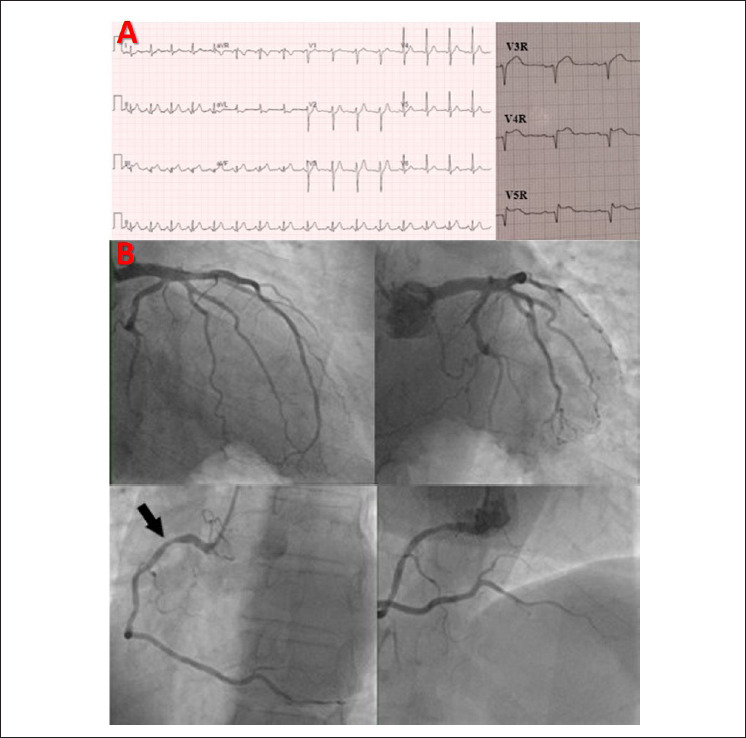
Painel A: O eletrocardiograma na admissão mostra supradesnivelamento do segmento ST nas derivações inferior e direita, bem como infradesnivelamento do segmento ST e inversão da onda T na derivação aVL. Painel B: Angiografia coronária mostra uma lesão não obstrutiva de 40% a 50% da artéria coronária direita proximal (flecha), com fluxo TIMI grau 3.

O eletrocardiograma mostrou ritmo sinusal e frequência cardíaca de 96 bpm, com supradesnivelamento do segmento ST nas derivações inferior e direita bem como infradesnivelamento do segmento ST e inversão da onda T na derivação aVL (Figura 1, painel A). Foi iniciada terapia dupla antiplaquetária e anticoagulante. Realizou-se angiografia coronária invasiva imediata, revelando uma lesão não obstrutiva de 40% a 50% da artéria coronária direita proximal, com fluxo TIMI grau 3 (Figura 1, painel B).

O ecocardiograma transtorácico na admissão não revelou alterações significativas, tais como anormalidades de movimento segmentar da parede, valvopatias, derrame pericárdico ou sinais de dissecção aórtica. Os ventrículos direito e esquerdo estavam dilatados e com função sistólica ventricular preservada (TAPSE 20 mm, velocidade sistólica do anel tricúspide 12,7 cm/s e fração de ejeção do ventrículo esquerdo 65%, pelo método biplano de Simpson). Os átrios direito e esquerdo não estavam dilatados (Material Suplementar).

Durante a internação hospitalar, o paciente permaneceu assintomático, sem recorrência da dor torácica, sintomas de insuficiência cardíaca ou arritmias documentadas por monitoramento eletrocardiográfico contínuo.

A análise laboratorial mostrou níveis elevados de troponina T de alta sensibilidade (valor máximo 1.790 ng/L, valor normal < 13 ng/L). As demais análises laboratoriais estavam dentro dos intervalos normais (NT-proBNP: 97 ng/L, D-dímero: 0,3 mg/L, hemoglobina: 14,1 g/L, leucócitos: 5.700, proteína C reativa: 0,2 mg/dL, creatinina: 0,9 mg/dL, AST: 71 UI/L, ALT: 35 UI/L, GGT: 49 UI/L, bilirrubina total: 0,6 mg/dL, TSH: 2,1 mU/L e T4 livre: 1,22 mU/L).

O eletrocardiograma realizado 2 dias após a admissão mostrou resolução das anormalidades observadas na admissão. Foi observada onda Q isolada na derivação DIII (Material Suplementar). A ecocardiografia transtorácica realizada 2 dias após a admissão não evidenciou anormalidades, tais como anormalidades na movimentação da parede ou disfunção do ventrículo direito.

**Figura 2:**
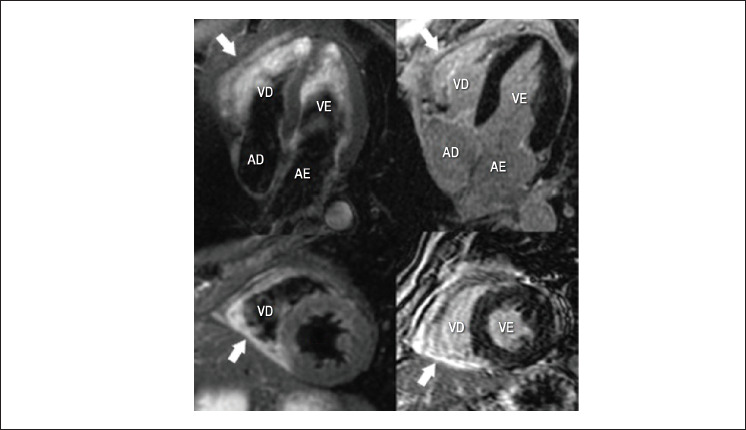
Diagnóstico de infarto do miocárdio isolado do ventrículo direito por imagens de ressonância magnética cardíaca. Nas imagens ponderadas em T2 (painel esquerdo), foi detectado aumento da intensidade do sinal na parede inferior do ventrículo direito, indicando edema miocárdico (flechas). Na análise de realce tardio com gadolínio (RTG) (painel direito), foi observado RTG na parede inferior do ventrículo direito (flechas), indicando a presença de necrose miocárdica. AD: átrio direito; AE: átrio esquerdo; VD: ventrículo direito; VE: ventrículo esquerdo.

Devido à presença de MINOCA, a ressonância magnética cardíaca foi realizada 4 dias após a admissão. As imagens de ressonância magnética cardíaca mostraram hipocinesia da parede inferior do ventrículo direito, com edema miocárdico nas imagens ponderadas em T2 e necrose miocárdica na análise de realce tardio com gadolínio (Figura 2). Foi estabelecido o diagnóstico final de infarto do miocárdio isolado do ventrículo direito.

## Discussão

O reconhecimento precoce do infarto do ventrículo direito em pacientes com infarto agudo do miocárdio é de importância primordial, não apenas para fins prognósticos, mas também porque pode orientar a terapia específica, incluindo a intervenção coronária percutânea primária agressiva, e evitar tratamentos que reduziriam ainda mais a pré-carga do ventrículo direito (nitratos e diuréticos), comprometendo o quadro do paciente.^4^^,^^5^

O diagnóstico dessa entidade é comumente realizado a partir de exame físico, eletrocardiografia, ecocardiografia e medidas hemodinâmicas.^5^

A tríade clássica observada durante o exame físico consiste em hipotensão, campos pulmonares limpos e pressão venosa jugular elevada.^6^

Deve-se suspeitar de infarto do miocárdio do ventrículo direito nos casos de infarto do miocárdio ínfero-posterior e realizar eletrocardiograma de derivação precordial direita, uma vez que a isquemia do ventrículo direito ocorre em até metade dos casos de infarto do miocárdio inferior.^4^^,^^5^

A ecocardiografia pode representar movimento anormal da parede livre do ventrículo direito e avaliar a presença de disfunção ou dilatação do ventrículo direito.^6^ As características adicionais de envolvimento do ventrículo direito incluem o movimento paradoxal do septo (interventricular e interatrial) e a presença de aumento do átrio direito ou regurgitação tricúspide.^6^

A ressonância magnética cardíaca pode ser útil para o diagnóstico, uma vez que é mais sensível que a eletrocardiografia e a ecocardiografia.^7^

A angiografia coronária leva ao diagnóstico final na maioria dos casos.^8^ O infarto do miocárdio do ventrículo direito ocorre principalmente devido à oclusão da artéria coronária direita proximal aos ramos principais do ventrículo direito no contexto de infarto do miocárdio inferior.^9^ Pode ocorrer também devido à oclusão da artéria circunflexa esquerda em pacientes com circulação dominante esquerda e, de forma menos frequente, em infartos anteriores, uma vez que a parede livre do ventrículo direito é fornecida por colaterais da artéria descendente anterior esquerda.^10^

Nosso caso de infarto do miocárdio do ventrículo direito isolado ilustra uma causa incomum de infarto do miocárdio. Foi único não apenas por se tratar de uma patologia rara, mas também por ter sido um desafio diagnóstico. O exame físico, a ecocardiografia e a angiografia coronária não foram capazes de estabelecer o diagnóstico final, visto que não apresentavam anormalidades significativas. Este caso enfatiza a importância da eletrocardiografia e o papel essencial da ressonância magnética cardíaca no diagnóstico diferencial de pacientes com MINOCA. Estabelecer o diagnóstico definitivo correto é de extrema importância para oferecer a terapêutica adequada, podendo ajudar a antecipar e prevenir complicações que variam de acordo com a etiologia.
