# Supplementary material for: Infarto Isolado do Ventrículo Direito – O Ventrículo Direito ainda é o Ventrículo Esquecido?
Source: Arq Bras Cardiol. 2021 Feb 2;116(2 Suppl 1):32–5. [Article in Portuguese] doi: 10.36660/abc.20200164 (PMC8118633; doi:10.36660/abc.20200164)
Supplement: Translation

# Translation: Isolated Right Ventricle Myocardial Infarction - Is the Right Ventricle Still the Forgotten Ventricle?

**Authors:** Ana Marques, Inês Cruz, Alexandra Briosa, Isabel João, Sofia Almeida, Hélder Pereira

**Affiliations:** 1 Almada Portugal Departamento de Cardiologia, Hospital Garcia de Orta, Almada - Portugal

**Keywords:** Myocardial Infarction, Coronary Artery Disease, Myocardial Revascularization, Percutaneous Coronary Intervention, Diagnosis Imaging

## Introduction

Isolated right ventricular myocardial infarction is extremely rare, and it is often silent, with only 25% of patients developing clinically evident hemodynamic manifestations on presentation.^1^ Current management of acute myocardial infarction is based on prompt diagnosis and immediate revascularization.^2^ About 90% of patients presenting with ST-segment elevation myocardial infarction have an explanatory coronary artery stenosis or occlusion.^3^ Myocardial infarction with non-obstructive coronary arteries (MINOCA) should lead the treating physician to investigate underlying causes, since failure to identify the underlying cause may result in inadequate and inappropriate therapy in these patients.

We describe a case of isolated right ventricular myocardial infarction with normal physical examination, transthoracic echocardiograms, and non-obstructive coronary artery disease on coronary angiography, whose definitive diagnosis was established by cardiac magnetic resonance imaging.

## Case Report

A 64-year-old white male was admitted to the hospital with a 1-hour history of sudden-onset acute oppressive anterior chest pain without other associated symptoms. After sublingual nitrate therapy, the patient presented total pain relief. His medical history included arterial hypertension, dyslipidemia, and former smoking.

At admission, the patient was conscious and hemodynamically stable (blood pressure: 130/70 mmHg and heart rate: 70 bpm), with apyrexy, eupnea, and peripheral oxygen saturation of 99%. No changes in cardiac and pulmonary auscultation were noted, and there was no elevated jugular venous pressure or edemas of lower extremities. Abdominal inspection was also normal.

Electrocardiography showed sinus rhythm and heart rate of 96 bpm, with ST-segment elevation in both inferior and right leads as well as ST-segment depression with T-wave inversion in lead aVL (Figure 1, panel A). Dual antiplatelet and anticoagulation therapy was started. Immediate invasive coronary angiography was performed, revealing a non-obstructive 40% to 50% lesion of the proximal right coronary artery, with TIMI grade flow 3 (Figure 1, panel B).

**Figure 1:** Panel A: Electrocardiography at admission showing ST-segment elevation in both inferior and right leads and ST-segment depression with T-wave inversion in lead aVL. Panel B: Coronary angiography showing a non-obstructive 40% to 50% lesion of the proximal right coronary artery (arrow), with TIMI grade flow 3.

The transthoracic echocardiogram at admission did not reveal significant changes such as segmental wall motion abnormalities, valvopathies, pericardial effusion, or signs of aortic dissection. Both left and right ventricles were non-dilated, and they had preserved ventricular systolic function (TAPSE 20 mm, tricuspid annular systolic velocity 12.7 cm/s, and left ventricular ejection fraction 65%, with the biplane Simpson method). Both right and left atria were non-dilated (Supplementary Material).

During hospitalization, the patient remained asymptomatic without recurrence of chest pain, heart failure symptoms, or arrhythmias documented by continuous electrocardiographic monitoring.

Laboratory analysis showed elevated high-sensitivity troponin T levels (maximum value 1,790 ng/L, normal value < 13 ng/L). The remaining laboratory analyses were within the normal ranges (NT-proBNP: 97 ng/L, D-dimer: 0.3 mg/L, hemoglobin: 14.1 g/L, leucocytes: 5,700, C-reactive protein: 0.2 mg/dL, creatinine: 0.9 mg/dL, AST: 71 UI/L, ALT: 35 UI/L, GGT: 49 UI/L, total bilirubin: 0.6 mg/dL, TSH 2.1: mU/L, and free T4: 1.22 mU/L).

The electrocardiography performed 2 days after admission showed resolution of the abnormalities noted at admission. An isolated Q wave was observed in lead DIII (Supplementary Material). The transthoracic echocardiography performed 2 days after admission did not show any abnormalities, such as wall motion abnormalities or dysfunction of the right ventricle.

Due to the presence of MINOCA, cardiac magnetic resonance imaging was performed 4 days after admission. The cardiac magnetic resonance imaging showed hypokinesis of the right ventricle inferior wall, with myocardial edema on T2-weighted images and myocardial necrosis on late gadolinium enhancement analysis (Figure 2). Final diagnosis of isolated right ventricular myocardial infarction was established.

**Figure 2:** Diagnosis of isolated acute right ventricular myocardial infarction by cardiac magnetic resonance imaging. On the T2-weighted images (left panel), increased signal intensity in the right ventricular inferior wall was detected, indicating myocardial edema (arrows). On late gadolinium enhancement (LGE) analysis (right panel), LGE was seen in the right ventricular inferior wall (arrows), indicating the presence myocardial necrosis. LA: left atrium; LV: left ventricle; RA: right atrium; RV: right ventricle.

## Discussion

The early recognition of right ventricle myocardial infarction in patients with acute myocardial infarction is of prime importance, not only for prognostic purposes, but also because it can guide specific therapy, including aggressive primary percutaneous coronary intervention, and avoid treatments that would further lower right ventricular preload (nitrates and diuretics), thus compromising the patient's condition.^4^^,^^5^

The diagnosis of this entity is commonly made from physical examination, electrocardiography, echocardiography, and hemodynamic measurements.^5^

The classic triad observed during physical examination consists of hypotension, clear lung fields, and elevated jugular venous pressure.^6^

Right ventricle myocardial infarction should be suspected in cases of infero-posterior myocardial infarction, and right precordial lead electrocardiogram should be performed, since right ventricular ischemia occurs in up to half of cases of inferior myocardial infarction.^4^^,^^5^

Echocardiography can depict abnormal movement of the right ventricular free wall, and it can assess the presence of right ventricular dysfunction or dilation.^6^ Additional features of right ventricular involvement include paradoxical motion of the septum (interventricular and interatrial) and the presence of right atrial enlargement or tricuspid regurgitation.^6^

Cardiac magnetic resonance imaging can be useful for diagnosis, because it is more sensitive than electrocardiography and echocardiography.^7^

Coronary angiography usually leads to the final diagnosis.^8^ Right ventricle myocardial infarction occurs mainly due to occlusion of the right coronary artery proximal to the major right ventricular branches in the context of inferior myocardial infarction.^9^ It may also occur due to occlusion of the left circumflex artery in patients with left-dominant circulation and, less commonly, in anterior infarctions, as the anterior part of the right ventricular free wall is supplied by collaterals from the left anterior descending artery.^10^

Our case of isolated right ventricle myocardial infarction illustrates an uncommon cause of myocardial infarction. Not only was it unique in being a rare pathology, but it was also a diagnostic challenge. Physical examination, echocardiography, and coronary angiography were not able to establish the final diagnosis, given that they did not show significant abnormalities. This case highlights the importance of electrocardiography and the essential role of cardiac magnetic resonance in the differential diagnosis of patients with MINOCA; establishing correct definitive diagnosis is of the utmost importance in order to provide appropriate therapy, and it can help to anticipate and prevent complications that differ according to the etiology.
